# Supplementary material for: A ten-year review of neonatal tetanus cases managed at a tertiary health facility in a resource poor setting: The trend, management challenges and outcome
Source: PLoS Negl Trop Dis. 2021 Dec 7;15(12):e0010010. doi: 10.1371/journal.pntd.0010010 (PMC8683026; doi:10.1371/journal.pntd.0010010)
Supplement: Attachment — Submitted filename: Response to Reviewers Comments NNT Manuscript.docx [file pntd.0010010.s003.docx]

**Response to Reviewers Comments**

We appreciate you for your prompt response to our submitted manuscript and we are grateful to the reviewers for the review of the manuscript and their comments which we believe has improved the quality of the manuscript.

These are our responses to the comments of the reviewers

The Title of the Manuscript: A Ten-Year audit of Neonatal Tetanus cases seen at Tertiary Hospital in a Resource Poor setting in Nigeria: The trend, management challenges and outcome.

The Title was changed according to the suggestions by the reviewers.

Methods

Reviewer #1: What is the socioeconomic background of Ekiti, the catchment area of EKSUTH. This information is important given the fact that tetanus is a disease of socioeconomic relevance. Information should be provided on how tetanus is managed in the unit. Any management protocol in place? What facilities in the management of neonatal tetanus and its complications are available in the centre. How was autonomic dysfunction diagnosed and managed?

**Response**

The socioeconomic background of Ekiti State and the catchment area of the institution have been included in the methodology section as well as the current treatment protocol as requested by the first reviewer. Also, information has been provided on how sepsis and autonomic dysfunction were diagnosed. Being a ten-year retrospective study, it is important to note that not all the detailed information about some of the cases may be available, however, the authors tried to go through the available records of all the patients and made use of all available information.

Since our facility is not equipped with ventilators, autonomic dysfunction was managed with the regular sedatives/ muscle relaxants that we use for tetanus patients in addition to giving analgesics occasionally.

Reviewer #1: Annual incidence rates should be plotted rather than the yearly distribution of absolute number of cases as shown in Figure 1. The incidence should be calculated as the number of cases in a year divided by the total admission for the year per 1000. That should be the emphasis in this study. The other aspects are at variance with the title of the manuscript and they add no new knowledge to the existing literature.

**Response**

The annual incidence rates have been plotted as suggested by the reviewers. Figure 1 now shows the annual incidence rate instead of the absolute number of cases that were used initially.

The title of the manuscript has been modified to indicate the intention of the authors which is to sensitize the policy makers to the need to intensify efforts at ensuring tetanus elimination in our country.

Reviewer #2: The analysis presented does not match the plan
The results are clearly presented but probably incomplete;
*All the babies were said to have umbilicus as the site without indicating how that was arrived at
*Table 1 - the aspect on cord care did not indicate if the methods of cord care were mutually exclusive
*Table 4 - What exactly is meant by age class
*What constitutes autonomic dysfunction was not shown, it was listed as a comorbidity whereas autonomic dysfunction is a manifestation of NNT
*What is referred to appropriate cord care was not defined
*About half were said to have had sepsis. How was sepsis established or ruled out?
*What the p value is about is unclear
*The figure on trend was expressed in absolute numbers without showing what proportion it is to total admissions. This may not depict the true picture with respect to trend in prevalence.

**Response**

We believe that the analysis now matches the aim and the objectives of the study.

We believe that the results are clear and complete now.

The umbilicus was the identified portal of entry in all our patients based on the findings when the babies were seen. There were features suggestive of hot water compress of the umbilical cord in the babies such as hyperaemia, some had exudates, and these were the documentation in the records of the patients when they were seen.

The cord care methods were not mutually exclusive, some mothers used more than one method of cord care. This has been indicated as a footnote below Table 1.

Table 4 – Age class has been changed to age group. The patients were grouped based on their age at presentation into less than one week and more than one week.

What constitutes autonomic dysfunction has been stated in the methodology section now. How this assessment was made has been included in the methodology section.

The authors agree with the comment that autonomic dysfunction is a manifestation of tetanus, however it is a manifestation of the severe form of the disease. The cell for that association has been modified as Complication/Co-morbidities. The goal of doing this was to further buttress the known fact that autonomic dysfunction is a poor prognostic feature of NNT.

The definition of appropriate cord care has been provided in the methodology section.

How sepsis was diagnosed has also been included in the methodology section of the manuscript.

The p-value was to test for significant association between some selected factors and the outcome of management of the babies.

The figure on the trend has been changed to the annual incidence as suggested by reviewer 1.

Reviewer #1: The conclusions need to be re-written when the focus of the study is re-addressed as suggested.

Reviewer #2: The conclusions are not adequately supported by the data presented
The discussion needs to focus on the aim of the study.

**Response**

The Conclusion has been re-written with the focus on the data presented in the result section. This now addresses the aim of the study.

Reviewer #1: This reads like a general overview on neonatal tetanus and should be reduced to a third of the current length. Studies focused on trend of the disease should be reviewed to suggest the dwindling incidence across Africa and Asia. This should replace the details of risk factors and clinical presentation as currently presented. Specifically, what is the trend in tetanus toxoid coverage across the geopolitical zones in Nigeria and in Ekiti State in particular. This will provide information on the success or otherwise of the Maternal and Neonatal tetanus Elimination Initiative
The tetanus toxoid coverage over the preceding five years should be discussed as it is one of the contributory factors to the dwindling incidence of tetanus in the newborn. There are reports that Traditional Birth Homes and Spiritual Birth Homes (Churches) who run antenatal clinics now liase with primary health centres for tetanus toxoid administration during pregnancy. The practice of unhygienic cord care might also have reduced. Relevant literatures from Southwestern Nigeria should be sought.

**Response**

We appreciate reviewer 1 for his comments, the study was a combination of general review on neonatal tetanus and its management challenges based on the practical experiences in our low resource setting. It also highlighted the state of things in our country when we ought to be talking about elimination. It is difficult to reduce the length to a third because of some further information requested by the reviewers, however, the introduction has been reduced. Most of the studies done in the country and some in Asia were included in the manuscript. One clear thing from the studies conducted in our country is the fact that the prevalence of NNT has reduced appreciably from what it used to be, but we are not yet there.
The general populace tetanus coverage does not directly impact on neonatal tetanus, rather it is maternal tetanus toxoid vaccination that positively reduce the burden of neonatal tetanus, and this has been addressed in the manuscript. The Maternal tetanus toxoid coverage over the past five years have been included in the manuscript. This is still not encouraging as shown in the manuscript.

Truly, TBAs and Spiritual Birth Homes (Churches) now liaise with primary health centres for tetanus toxoid vaccination, but the unfortunate thing is that most of the mothers in this study did not take the tetanus toxoid vaccine. This practice started not long ago, and this study is a ten-year retrospective study most likely when this has not commenced. The other challenge is that this practice is not documented in any manuscript for referencing.

The practice of unhygienic cord care has not reduced based on our practical experience and interaction with mothers. Most of the traditional methods of cord care are still being employed by mothers in our area. Unfortunately, there is no recent literature to support this.

Reviewer #1: This is a retrospective study from a single centre in Nigeria. Remarkably, the cases presented are quite few and that made the statistical analysis bogus. The focus of the study is at variance with the title of the manuscript. The title suggests a trend analysis but the body of the manuscript dwells more on the clinical presentations of the disease. Going by the current title, a comparison of the incidence and case fatality rates at EKSUTH should be compared with similar data obtained from other centres across Nigeria over the same period of time. The data from a single centre will not be enough for generalization as the title suggests. Otherwise, the title should be amended to focus only on EKSUTH.

**Response**

The authors agree with the submission of this reviewer that the focus of the study was at variance with the initial Title. The Title has been amended as advised and the manuscript reflects the title now.

Reviewer #2: The topic is of public health importance.
I suggest modifications to the title as the study design and the result presented cannot answer the question posed in the title.
This is only a review of NNT in one hospital in one geopolitical zone of the country. Even the yearly trend for the hospital requires more information on the proportion relative to other causes of neonatal admissions which may be affected by several factors. it can therefore not be extrapolated to the whole country as the study was not powered for such.
Introduction, paragraph 2 - other possible portal of entry of tetanus deserve to be mentioned
Paragraph 3, 1st sentence in introduction should be referenced, same with paragraph 5.

Response

The title has been modified.

Other portal of entry of tetanus have been mentioned as requested.

The sentence has been referenced

The sentences have been referenced as suggested by reviewer 2.

Once again, we appreciate the reviewers for their comments which we believe have made the manuscript richer and better.
